# Are All Urban Parks Robust to the COVID-19 Pandemic? Focusing on Type, Functionality, and Accessibility

**DOI:** 10.3390/ijerph19106062

**Published:** 2022-05-16

**Authors:** Hyungun Sung, Woo-Ram Kim, Jiyeon Oh, Samsu Lee, Peter Sang-Hoon Lee

**Affiliations:** 1 Department of Urban and Regional Development, Graduate School of Urban Studies, Hanyang University, 222 Wangsimni-ro, Seongdong-gu, Seoul 04763, Korea; hgsung80@hanyang.ac.kr (H.S.); pheonixw@naver.com (W.-R.K.); ellawinella@gmail.com (J.O.); 2 Land and Housing Institute, Daejeon 34047, Korea; l3water@lh.or.kr

**Keywords:** mobile phone data, multi-level regression model, park type, physical feature of urban park, urban park visit

## Abstract

Many people visited urban parks during the COVID-19 pandemic to reduce the negative effects of lack of physical activity, social isolation, anxiety, and depression. It is unclear whether all parks are robust against the pandemic, helping people sustain healthy daily living through the diverse activities within them. Nevertheless, few studies have identified the specific relationship between park visits and the COVID-19 pandemic. Therefore, this study aims to demonstrate how physical features such as type, functionality, and access influenced daily visiting to parks during the pandemic, using mobile phone data at a micro level. This study first classified urban parks as point-type parks with an area of less than 1 ha, plane-type parks with 1 ha or more, and line-type parks with elongated shapes, while measuring accessibility to residential, employment, transportation, and auxiliary facilities within the park. The study employed the multi-level regression model with random intercept to investigate the effects of differing park visits, focusing on Goyang city, South Korea. Our analysis results identified that easy access from home was more important than the park size during the pandemic. If we look at the types of parks, the use of both plane- and point-type parks increased more than that of line-type parks. However, line-type parks near homes, along with shopping and sports facilities, were found to be more robust to the pandemic. These findings can be informative to provide specific guidelines to fulfill the enhanced role of parks in sustaining public health during an infectious disease pandemic that may strike again.

## 1. Introduction

First reported in Wuhan, China in December 2019, COVID-19 has caused more than 211 billion confirmed cases and more than 4.42 million deaths worldwide as of 22 August 2021. At the same period of time, South Korea and Seoul recorded 236,366 and 74,749 confirmed cases and 2215 and 571 deaths, respectively. Accordingly, almost all cities around the world have implemented various non-pharmaceutical interventions such as social distancing, movement restrictions, and the closure of workplace and indoor recreational facilities to reduce its spread, especially prior to the dissemination of the vaccine. However, even if such measures have been effective in suppressing the spread of the disease, unexpected negative public health effects such as lack of physical activity, social isolation, and anxiety and depression due to the lack of social interaction might also appear [1]. Recent research has reported that people across the world visited more green spaces such as parks and urban forests during the COVID-19 pandemic to reduce these negative effects [2,3,4,5,6,7]. The increased visits have been observed because of the positive effects of urban parks on psychological and physical well-being, social cohesion, and mental wellness [1,8,9,10,11,12,13,14,15,16,17].

During the pandemic, urban parks may have robust properties, providing a pathway for exercise, leisure, and social interactions for people with limited mobility. In particular, urban parks, which can be easily accessed by citizens, can play an important role in sustaining and regaining a healthy lifestyle in response to the COVID-19 pandemic [13,15,18,19]. Urban parks, including green, urban forests, and/or open spaces within urban areas, including riparian land, may have a positive influence in terms of providing rest areas, socializing spots, and improving the quality of health and wellbeing [20,21,22]. Different features, such as the size, location, and purpose of urban parks, can lead to very different outcomes, even in a similar urban area. In general, it is expected that the larger the urban parks, the greater the benefits provided to the users. In addition, research has indicated that relatively small parks with abundant trees and flowers could exert a positive influence on park visitors [23,24,25]. However, there has been a lack of studies dealing with urban parks in simplified and standardized forms by size and shape or addressing the effects of facilities provided by urban parks.

However, the question whether all urban parks are robust as a safe and available open space against the COVID-19 pandemic should be answered. It is significant to make relevant policy decisions for better planning of urban parks as a measure against an infectious disease pandemic that may strike again. It is well known that the use of urban parks was context-dependent in normal life before the COVID-19 pandemic took place [20,21]. Therefore, it is meaningful to understand how urban park visits during the COVID-19 pandemic were affected by attributes such as type, function, and accessibility. Few studies have identified a specific relationship between urban park visits and the COVID-19 pandemic. Using mobile phone data at a micro level, this study aims to demonstrate how the types and attributes of urban parks, such as their size, shape, functionality, and accessibility, influenced the visits they received during the recent pandemic. We employed the multi-level random intercept model to investigate the effects of differing urban park visits, focusing on Goyang city, South Korea. The findings of our study can have policy implications for the location and placement planning of urban parks, so that both people and cities can benefit during an infectious disease pandemic.

## 2. Materials and Methods

### 2.1. Measurement

**Figure 1:**
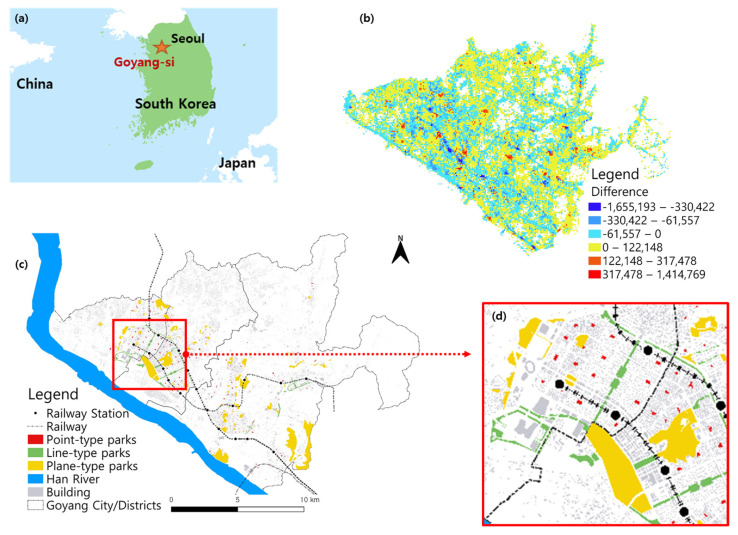
(**a**) Location of the study area, Goyang City, near Seoul, South Korea; (**b**) Distribution of the increase/decrease in the floating population affected by COVID-19 pandemic within the study area; (**c**) Distribution of the locations of urban parks within the study area showing the different types of urban parks on a clear display; (**d**) An enlargement of part of Figure 1.

This study aimed to empirically identify which urban parks in South Korea were visited more during the COVID-19 pandemic compared to the pre-pandemic period. The study area was Goyang city in Gyeonggi-do, located outside the northwestern part of Seoul, the capital of South Korea (Figure 1a). The number of confirmed COVID-19 cases in Goyang city was 1797, which was the highest among municipalities, except the district of the Daegu metropolitan city (1925) in South Korea, as of 18 January 2021. The study area was developed as a new city in the early 1990s to accommodate the explosive population growth in Seoul. Its population of 257,654 in 1992 grew tremendously, making it a metropolis spread out over an area of 267.3 km^2^ with 1,081,045 residents as of May 2021. Goyang is closely dependent upon Seoul, with many inhabitants commuting to school and work, especially 32.4% of the population aged 15 years and older.

Mobile phone data could be useful for effectively monitoring non-pharmacological interventions during the COVID-19 pandemic, evaluating the spatiotemporal spread of potential confirmed cases, and supporting contact tracing [26,27]. The original data were collected by the Korean telecommunication company SKT. The data on the number of communications (calls, text messages, etc.) were estimated in units of x-and y-coordinates at intervals of 50 m × 50 m. This study measured the number of daily visitors to urban parks using information on the location trajectory of mobile phone data derived from call logs at a micro level of a 50 m × 50 m grid cell. Figure 1b illustrates the spatial distribution of the difference in the mobile-based floating population in the study area before and during the pandemic on a grid cell scale. The floating population was measured using location-based mobile-phone data.

Figure 1c presents the spatial distribution of 383 urban parks in the study area. A total of 2512 grid cells were identified to be within urban parks among the approximately 200,000 cells covered by the study area during our study period. Table 1 shows the summary statistics of the average daily park visits at both the cell and park levels considering part type, relevant facility, and transportation mode for two periods before and during the COVID-19 pandemic by grid cells. One period is for the 1-year term before the COVID-19 pandemic (from 1 February 2019 to 31 January 2020), and the other is for the first year during the pandemic (from 1 February 2020 to 31 January 2021). Prior pandemic data were employed in the study to serve as a control treatment in order to identify the pure impact of the pandemic on visits to urban parks. The average number of daily urban park visitors for the two periods and the difference between the two periods were examined. The average number of daily visitors per cell before and during the pandemic for one year were 332.9 and 336.0, respectively.

By reviewing a range of relevant studies, various forms of urban parks were classified into three categories: point, plane, and line. Based on the size of the urban park, a certain group of parks smaller than 1 ha was defined as point-type parks, and parks equal to or larger than 1 ha were defined as plane-type parks. Elongated parks generally along roads and/or streets, such as tree-lined streets, green corridors, and/or parkways, were defined as line-type parks, not by size but by shape. According to this classification, children’s parks and neighborhood parks in South Korea are regarded as point-type parks, and parks larger than 1 ha are regarded as regional neighborhood parks [26]. The purpose of line-type parks in this study included a movement route along with traditional park functions, such as rest, strolling, and exercise. Pan et al. [28], however, reported that a highly connected linear green space inferred from a geo-spatially varying network-based risk model has a high probability of confirmed infectious diseases. Plane-type parks accounted for 55.5% of the total number of urban parks in the study area, followed by point −(23.5%) and line-type (21.1%) parks.

The urban park visits varied depending upon the type of facilities within the park, as well as its type. That is, the use of parks is context-dependent [29]. Visitors to the park mainly use it as a place for activities such as walking and resting, as well as moderate-intensity physical activity [30]. Therefore, this study measured the context within the park, or the facilities it provided. Urban parks within the study area contained sports facilities such as outdoor gymnastics (39.8%), play facilities such as playgrounds, swings, and seesaws (17.3%), and cultural facilities such as small libraries and outdoor music halls (6.8%).

Accessibility to urban parks may affect the visits received during the COVID-19 pandemic. Huerta and Cafagna [31] confirmed that a lack of access to urban green spaces prevented people from visiting them during the COVID-19 pandemic. In addition, visitors travelling with their own vehicles may be dependent on the difference in the perception of the COVID-19 vulnerability of each transport mode. Abdullah et al. [32] and Shakibaei et al. [33] demonstrated that changes in travel behavior, such as less use of public transit and a shift to a private car for movement occurred during the COVID-19 pandemic.

The accessibility of private cars, buses, and subways were measured by using a buffer concept [34]. Accessibility by private cars was measured using the presence of a parking lot within the park. Urban parks with parking areas occupy 35.3% of the total number of cells in this study. The accessibilities of buses and subways were measured by confirming the number of stations within a 100 m radius buffer on a grid cell and park boundary basis, respectively. Because many park visitors are dependent on public transportation, people’s visits to parks are, in general, strongly related to using those transport modes. Within the grid cell buffer of 100 m radius, on average, the number of existing bus stops was 0.415. Grid cells with subway stations located within a radius of 100 m accounted for only 0.4% of the total park grid cells. In addition, within 100 m of the park boundary, the number of bus stops present was 6.035, and subway stations existed in 6.80% of the total park grid cells.

Another accessibility indicator that affects park visits was the presence of a large shopping mall, which tends to induce a floating population in the immediate vicinity of the park. Visitors to these facilities are more likely to visit the park before and after visiting the facility if the two are located close to each other. In this regard, this study measured whether these facilities were located within a radius of 100 m from the boundary of each cell and park. The proximity to shopping malls was 3.60% by cell and 19.2% by park boundary.

Park visits may depend on both the physical environmental attributes of the surrounding area and the proximity of the park. A study by van Vliet et al. [25] found that parks were visited by people located within 1 km radius. Wolch et al. [35] reported that the distance that could be accessed by walking for a park visit was within the 500 m boundary. Poortinga et al. [13] also reported that subjective health and well-being efficacy decreased when a public green space took more than 10 min on foot. The Korean Urban Park Law stipulates that the threshold distance of urban parks is 500 m for children and neighborhood parks and 1000 m for metropolitan parks [36]. Donahue et al. [37] demonstrated that population density near parks could positively influence visitation. Therefore, this study measured physical environmental attributes within a radius of 500 m and 1000 m from the boundary of the park. The attributes of the neighboring physical environment included daily neighborhood facility density (m^2^/km^2^), the presence of a general hospital, population density (persons/km^2^), and employment density (persons/km^2^). In our preliminary analysis, the 500 m physical environment attributes were more statistically significant compared to those in the 1000 m buffer. Therefore, the limited distance of the physical environmental attributes around the park was employed in the final model. The averages of daily neighborhood facility density (m^2^/km^2^), population density (persons/km^2^), and employment density (persons/km^2^) within a radius of 500 m were 2852.5, 2991.8, and 2409.98, respectively. Proximity to a general hospital was 33.8% by park boundary.

The zoning type may also affect the average daily parking visits. For example, Sung et al. [38] demonstrated that walking activity on the street differed depending on the type of zoning. Since urban parks are closely related to pedestrian activities, the number of park visits may vary depending on the designated zoning type around the urban park. The zoning type was classified into residential areas (41.3%), commercial areas (6.3%), green areas (44.6%), and others (7.8%).

The city’s population has grown continuously since the early 1990s. Therefore, an increase in population during the analysis period may affect visits to urban parks. The population growth during the analysis period in the administrative district where the parks were located was measured. The increased population before and during the pandemic and the difference between the two were 1754.8, 786.8, and 14,752.7, respectively.

### 2.2. Methodology

It was assumed that the average number of daily urban park visitors ($y_{ij}$) based on the mobile trajectory data in a 50 m × 50 m grid cell (*i*) located in park (*j*) was affected by the predictors of both the cell ($X_{ij}$) and park ($X_{j}$) criteria. A park can have several 50m grid-based cells. In Table 2, the number of urban parks is 338 and the number of grid-based cells is 2512. Therefore, a park will have 7.43 grid cells on average in the study. A multiple linear regression model was used first, allowing us to estimate the outcome variable ($y_{ij}$), number of visitors in the grid-cell unit (*i*) within the park-level unit (*j*), affected by predictors $X_{ij}$ with grid-cell unit (*i*) within the park (*j*), and $X_{j}$ only park-level (*j*). This two-level regression model, also called a random effects model, is more suitable for data from *j* parks with a different number of grid cells (*i*). Therefore, a multilevel random model dealing with the grid-cell level (*i*) and park level (*j*) was developed.

$$y_{ij}=a_{ij}+\beta_{ij}X_{ij}+\beta_{j}X_{j}+u_{oj}+\varepsilon_{ij}$$

This equation indicates that the average number of daily park visitors ($y_{ij}$) at the grid-cell level (*i*) within a park level (*j*) is determined by the attributes $X_{ij}$, of the grid level (*i*) within the park group level (*j*) and $X_{j}$ only at the park level (*j*). It is assumed that the random intercept ($u_{oj}$) and the residual ($\varepsilon_{ij}$) in this hierarchical model are mutually independent. If these data are analyzed as an ordinary least square linear regression model with a single level, bias can be induced because of ecological fallacy [38,39].

A total of three two-level linear regression models were developed. Our key dependent variables in the first and second model (Models A and B) were the average number of daily urban park visitors during the one year before the pandemic and for one year after that, respectively. The third model (Model C) was used to empirically examine the effect of a number of independent variables on the difference in the average number of daily urban park visitors before and during the pandemic. In all three models, the dependent variables were treated in a log-transformed form. The log model indicates the % change in the number of urban park visitors with a one-unit change in the independent variable.

## 3. Results

### 3.1. Model Diagnoses

The bottom part of Table 2 summarizes the statistics of fitness and reliability diagnoses for each model. First, in this study, the fitness diagnosis of the multilevel random intercept linear regression model over the ordinal least squares linear regression model was conducted. The adjusted R-squared statistics for the one-level linear regression models were 0.48 for Model A, 0.43 for Model, B, and 0.05 for Model C. In multilevel regression models, the marginal *R*-squared indicates only the variance of the fixed effects, whereas the conditional *R*-squared considers both fixed and random effects [40]. The marginal *R*-squared statistics were 0.348 for Model A, 0.295 for Model B, and 0.102 for Model C, whereas the conditional R-squared statistics were 0.518, 0.478, and 0.614 for Models A, B, and C, respectively. The conditional *R*-squared statistic for each model has a higher value than the marginal *R*-squared statistic and the adjusted *R*-squared statistic. This indicates that the multilevel regression models with both fixed and random effects performed better in our analyses.

The diagnosis statistic was the Akaike information criterion (AIC). In general, a model with a smaller AIC value is a more suitable model than that of a larger AIC value. Table 2 indicates that the AIC statistics for the multi-level linear regression model were lower than those of the one-level regression model. Thus, this study identified that the multi-level regression model was more suitable than the ordinary least square (OLS) regression model for each dependent variable. All three model statistics in this study indicated that the variation in both random and fixed effects was larger than the variation in the only fixed effect. This implies that a model that considers the random effect can be more suitable.

Second, the reliability of the models was diagnosed using intra-class correlation (ICC) statistics [39,41,42]. The ICC was calculated by dividing the total variance by group, i.e., the inter-park variance. This was an indicator of how large the variance in the number of urban park visitors among the high-level groups was compared to the overall variance. The ICC values of the unconstrained models were estimated using only the intercept, without all the independent variables. The values for Models A, B, and C were 0.403, 0.382, and 0.618, respectively (Table 2). In particular, the difference in the number of park visitors changed due to the pandemic (Model C) was much larger than that of Models A and B. In other words, the difference in the number of park visits due to the pandemic would be affected more by higher-level park attributes than by cell-level ones. On the other hand, the ICC values of Models A, B, and C, including all these independent variables, were 0.348, 0.295, and 0.57, respectively, which are smaller than those of the null model. This implies that the variance in the dependent variables decreased as they were explained by the independent variables included in the model.

### 3.2. Results of Regression Analyses on Urban Park Visits

First, compared with the pre-pandemic period, the number of park visitors per cell increased by 3.1 individuals during the pandemic in Table 1. This implies that people visited urban parks to cope with the risks of mental health, such as anxiety, depression, and stress, as well as physical health issues caused by non-pharmaceutical measures such as movement restriction and social distancing due to the COVID-19 pandemic [2,7,17,31].

By comparing Model B to Model A in terms of statistical significance and the magnitude of the regression coefficients, it was evident that the average number of daily park visits during the pandemic was differentiated through the change in the influence power of the predictors. First, there was little difference in the statistical significance between the pandemic period and the pre-pandemic period, except in the cases where cultural facilities existed in the park and a high number of bus stops were present around it. Unlike Model A, the results from Model B indicated that the number of visitors during the pandemic decreased when urban parks had cultural facilities or more bus stops around it. This might have been due to avoidance behavior of people toward facilities with a relatively high risk of spreading COVID-19.

Second, this study identified that all statistically significant regression coefficients in Model B decreased in Model B as compared to those in Model A. This implies that the influence of the predictors on the number of average daily visitors to urban parks has become smaller due to the pandemic. Third, by comparing the size of the constant term of the two models, it was confirmed that attributes other than the predictors that had a significant effect on park visits influenced its variation. The constant terms for Models A and B were 4.19 and 4.27, respectively. The constant term in both models means that the average number of daily visitors to the park increased on average in Model B (during the pandemic) compared to that of Model A. That is, during the pandemic period, the average daily park visits increased by 0.08% (=4.27 − 4.19), while controlling for the attributes of the determinants included in the model.

What other attributes influenced the difference in the number of average daily visitors to urban parks during the COVID-19 pandemic? This was confirmed by the results of Model C. In Model C, the statistically significant variables among the attributes influencing park visits were the line-type park, the number of bus stops located within a 100 m radius of the grid cell, and the population and employment density within a 500 m radius from the park boundary. Among the types of urban parks, visits to line-type parks decreased by 0.1148%. This means that if an urban park was also a major movement route for travel purposes other than its original function, such as rest, the number of uses during the pandemic decreased. The size of the park was not statistically significant, as the difference in the number of daily visits to the park in Model C was similar to the results of Models A and B.

On the other hand, the results of Model C in Table 2 show that as the number of bus stops within a 100 m radius of the grid cell increased, the use of the park site decreased by 0.027%. This demonstrates that the use of certain places within a park tended to decrease in places where anonymous people tend to gather, such as condensed bus stops, because of the relatively high risk of COVID-19 spread.

The results of Model C also showed that the visits to urban parks increased as the number of people living around the park increased, while its visits decreased as the number of working people around it increased. This can be due to policy measures such as restrictions on movement by the quarantine authorities and recommendations to stay at home and work from home during the COVID-19 pandemic. In other words, as more people stayed at home instead of at work during the pandemic, the demand for parks near their homes increased, while the demand for parks near work decreased.

## 4. Discussion

Green spaces such as parks, rivers, and open spaces not only serve as a place for resting and social interaction but also have a positive impact on the quality of life and health of people [43]. This study contributes to landscape and urban planning by demonstrating that the role of urban parks has become more important during the COVID-19 pandemic. This is because green areas, including parks, have emerged as places to sustain the quality of life of people while responding to policy interventions or controls, such as movement restrictions and social distancing. For example, greenness has been shown to lower the number of confirmed cases and deaths of COVID-19 in cities with high population densities [44]. Urban parks can be more important because they are located closer to homes and can be more closely and easily linked to daily life [1,2,13,15,18,19]. The number of visitors to urban parks increased significantly during the COVID-19 pandemic [5,6,7,17]. Volenec et al. [7] found that visits to parks in New Jersey, USA, increased by 63.4%. Our study also supports the idea that more people visited urban parks due to their enhanced positive role during the pandemic. The number of park visitors per cell during the pandemic increased by 3.1 individuals, compared with the pre-pandemic period. From our analysis results, as well as other cases [7], urban parks may have an important role in managing the risks to mental health as well as physical health during the COVID-19 pandemic [2,17,31].

However, the conclusions of previous studies about the relationship between the robustness of urban parks with respect to COVID-19 and their size have been inconsistent. Recent studies have revealed that the efficacy of relatively small neighborhood parks is greater during the pandemic [5,9,25]. Park et al. [5] found that the use of small neighborhood parks in residential areas increased by 3% to 6% during the COVID-19 pandemic. On the other hand, Huerta and Cafagna [31] found that residents of Mexico City visited more parks during the pandemic if it was nearby, regardless of its size. This study proved that the size of the park might not be important, but its type is significant in determining whether people visited it. Visits to line-type parks decreased in comparison to plane-type or point-type parks during the pandemic. This supports the argument that COVID-19 infection is associated more with highly connected green spaces in London, UK [28].

**Figure 2:**
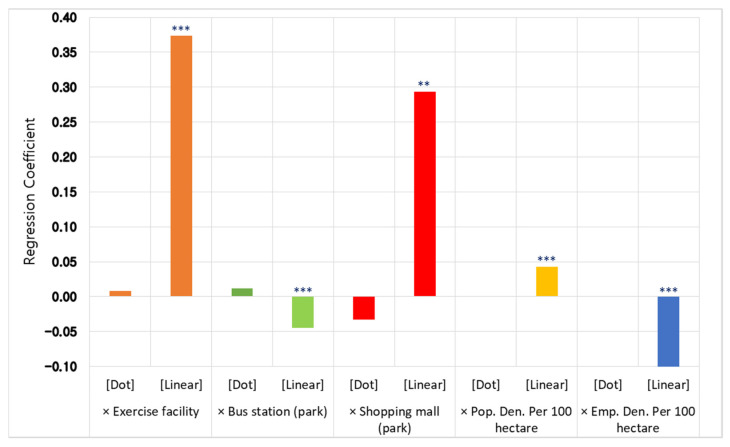
Regression coefficients for interaction terms by park type compared to the plane-type parks as a reference. (**: *p* < 0.01; ***: *p* < 0.001; [Dot] for point-type parks and [Linear] for line-type parks).

Then, do people refrain from visiting all line-type parks? Our study reveals that the answer is “no”. This was proven through additional analysis of the interaction terms between the park type and the physical environment with and without the park in Model C (Figure 2). More people visited line-type parks such as parkways which had outdoor sports facilities within it, as well as shopping facilities and more resident populations nearby. Our findings are similar to those of previous studies that indicated more people visited the park for exercise [30,45]. This study can be useful for policymakers and planners, as it proves that more people used the park during the pandemic when sports facilities were installed in a line-type park. Based on our results, a more specific policy guideline can be created compared with the results of previous studies. For example, a linear city park could play a strong role during the epidemic by arranging outdoor sports facilities near residences and commercial areas, rather than serving only as a passage for human movement.

Findings from this study demonstrated how the types and attributes of urban parks influenced visits during the recent COVID-19 pandemic based on mobile phone data at the micro level. Many recent studies may have limitations because they identified the role of urban parks based on online surveys conducted during the pandemic [1,11,15,45]. However, our study did not identify the difference in the number of confirmed cases during the period of COVID-19 and the differential effect on park visits by type of quarantine measures. Volenec et al. [7] and Tyrovolas et al. [3] reported the effect of COVID-19 policies on park visits. Veitch et al. [30] also identified that the nature of park visits varies by gender and age group. We can expect that the moderating effect of park visits depending on park type and attributes may also be differentiated according to non-pharmaceutical intervention measures and groups of users during the COVID-19 pandemic.

It is valuable to empirically identify the robustness of urban parks during the COVID-19 pandemic by their types, functions, and accessibility using multilevel regression modeling. Nonetheless, this study had some limitations. First, it did not demonstrate that the robustness of urban parks was consistent over time during the pandemic, as this may have been affected by non-pharmaceutical interventions that limited human mobility during the COVID-19 pandemic. Therefore, it is necessary to further study temporal changes in the effectiveness of urban parks for these interventions. Another limitation is that this study focused only on urban parks. Other types of greenness, such as rivers and open spaces, may play an important role in sustaining healthy human lifestyles. Therefore, we need to investigate their robustness during the COVID-19 pandemic. 

## 5. Conclusions

This study is considered useful in the field of landscape and urban planning in that it provides specific guidelines to fulfill the enhanced role of urban parks in sustaining public health during an infectious disease pandemic that may strike again. The study reveals that easy walkability from home is more important than the size of the park. Our findings indicate that small nearby parks can be more important to people, especially in already-urbanized cities where there is no more space available to develop large new ones. Our research findings also demonstrate that it is more desirable to improve existing parks by adding more attractive facilities, such as space and equipment for exercise. Among the types of urban parks, the use of both plane- and point-type parks has increased compared to that of line-type parks. However, line-type parks near homes and with shopping or sports facilities were more robust during the pandemic, even though they may be more susceptible to the spread of COVID-19. Easy access to and use of urban parks was found to be positively correlated with life satisfaction, subjective health, and well-being during the COVID-19 pandemic [9,11,13,18]. Therefore, our findings have policy implications, as they can help plan the location and placement of parks so that both people and cities can become more robust during an infectious disease pandemic.

## Figures and Tables

**Table 1:** Summary statistics.

Variable			No. of Grid	Average (Percent)	Std. Dev.	Min	Max
Dep. Var.	Average daily park visit before pandemic (A)		2512	332.9	636.671	0	10,494.6
	Log-visitor before pandemic (Log A)		2512	4.1	2.349	0	9.3
	Average daily park visitor during pandemic (B)		2512	336.0	593.846	0	8324.6
	Log-visitor during pandemic (Log B)		2512	4.3	2.188	0	9.0
	Difference in park visiting (C = A − B)		2512	3.1	180.257	−2170.1	1565.9
	Log-difference (Log (C + min(C)))		2512	7.7	0.187	−0.1	8.2
Independent Variables	Park type	Plane (ref.)	1394	55.5%			
		Line	529	21.1%			
		Point	589	23.5%			
	Exercise facility	No (ref.)	1513	60.2%			
		Yes	999	39.8%			
	Play facility	No (ref.)	2078	82.7%			
		Yes	434	17.3%			
	Cultural facility	No (ref.)	2340	93.2%			
		Yes	172	6.8%			
	Parking lot	No (ref.)	1626	64.7%			
		Yes	886	35.3%			
	No. bus stations (cell)		2512	0.4	0.841	0	6
	Subway station (cell)	No (ref.)	2503	99.6%			
		Yes	9	0.4%			
	Shopping mall (cell)	No (ref.)	2422	96.4%			
		Yes	90	3.6%			
	No. bus stations (park)		2512	4.7	6.035	0	25
	Subway station (park)	No (ref.)	2340	93.2%			
		Yes	172	6.8%			
	Shopping mall (park)	No (ref.)	2029	80.8%			
		Yes	483	19.2%			
	Daily neighborhood facility density (m^2^/km^2^) within 500 m buffer		2512	2852.5	6017.612	0	47,259.0
	General hospital within 500 m buffer	No (ref.)	1664	66.2%			
		Yes	848	33.8%			
	Population density (persons/km^2^) within 500 m buffer		2512	2991.8	4055.741	45.4	25487.1
	Employment density (persons/km^2^) within 500 m buffer		2512	2410.0	2492.544	14.6	13,904.5
	Zoning	Commercial (ref.)	158	6.3%			
		Green	1121	44.6%			
		Residential	1037	41.3%			
		Others	196	7.8%			
	Difference of population before pandemic (Model A/Model B/Model C)		2512	1754.8/786.8/14,752.7	4207.8/2190.6/37702.5	−1192/−1029/−14821	13,973/7310/126,799

**Table 2:** Analysis results on regression models.

Predictors		Model A: Before Pandemic			Model B: During Pandemic			Model C: Difference		
		Estimates		Std. Error	Estimates		Std. Error	Estimates		Std. Error
(Intercept)		4.19416	***	0.24774	4.26814	***	0.23985	7.7047	***	0.0379
Park type [Point]		0.91456	***	0.19956	0.81039	***	0.19378	0.011		0.0345
Park type [Line]		0.66161	*	0.25532	0.59893	*	0.24819	−0.1148	*	0.0454
Exercise facility [Yes]		0.47204	**	0.15845	0.46162	**	0.15395	0.0381		0.0268
Play facility [Yes]		0.31202	+	0.16818	0.30173	+	0.1639	0.0129		0.0276
Cultural facility [Yes]		−0.72998		0.4641	−0.79357	+	0.45045	0.099		0.0865
Parking lot [Yes]		−0.14809		0.22628	−0.10215		0.21983	−0.0217		0.0408
No. bus stations (cell)		0.64782	***	0.04154	0.59071	***	0.04038	−0.0266	***	0.0045
Subway station (cell) [Yes]		0.10208		0.53954	−0.10459		0.52452	−0.0398		0.0568
Shopping mall (cell) [Yes]		0.64434	***	0.18485	0.49374	**	0.17971	−0.0057		0.0194
No. bus stations (park)		0.04067	+	0.0227	0.03575		0.02205	0.0027		0.004
Subway station (park) [Yes]		−0.58639		0.45308	−0.48451		0.43999	0.0287		0.0796
Shopping mall (park) [Yes]		−0.25423		0.29878	−0.11511		0.29019	0.0865		0.053
Daily neighborhood facility density (m^2^/km^2^) within 500 m buffer		0.00001		0.00001	0.00000		0.00001	0.00000		0.00000
General hospital within 500 m buffer [Yes]		0.31797	*	0.15787	0.35964	*	0.15263	−0.0072		0.0263
Population density (persons/km^2^) within 500 m buffer		0.00001		0.00002	0.00002		0.00002	1.051 × 10^−5^	**	3.139 × 10^−6^
Employment density (persons/km^2^) within 500 m buffer		0.00006		0.00004	0.00006		0.00004	−3.295 × 10^−5^	***	6.139 × 10^−6^
Zoning [Green]		−1.0279	***	0.16338	−0.83993	***	0.15856	0.0013		0.0176
Zoning [Others]		−1.04095	***	0.21447	−0.85381	***	0.20842	0.0217		0.0243
Zoning [Residential]		−0.56588	***	0.1586	−0.52325	**	0.15405	0.005		0.0171
Difference of population		−0.00006	***	0.00001	−0.00005	*	0.00002	0.00000		0.00000
Random Effects										
$\sigma^{2}$(Variance at the park level)		2.13			2.01			0.02		
τ (Variance of the intercept at the unit level)		0.75			0.70			0.03		
$ICC_{full\_model}$ $/ICC_{null\_model}$		0.26/0.403			0.26/0.382			0.102/0.614		
Model statistics	Observations at the park level	338			338			338		
	Observations at the cell level	2512			2512			2512		
	Marginal R^2^/Conditional R^2^	0.348/0.518			0.295/0.478			0.102/0.614		
	AIC	9382.24			−1705.093			−1705.093		
OLS model statistics	Adjusted R^2^	0.48			0.43			0.051		
	AIC	9801.309			9693.138			−1390.727		

Note: *p*-value < 0.001, “***”; *p*-value < 0.01, “**”; *p*-value < 0.05, “*”; *p*-value < 0.1, “+”.

## Data Availability

Data used in this study are available from the authors upon reasonable request.
